# Supplementary material for: Single-cell RNA sequencing of the holothurian regenerating intestine reveals the pluripotency of the coelomic epithelium
Source: bioRxiv. 2024 Dec 31:2024.07.01.601561. Originally published 2024 Jul 4. Preprint. [Version 2] doi: 10.1101/2024.07.01.601561 (PMC11244903; doi:10.1101/2024.07.01.601561)
Supplement: 1 [file NIHPP2024.07.01.601561v2-supplement-1.pdf]

bioRxiv preprint doi: https://doi.org/10.1101/2024.07.01.601561; this version posted December 31, 2024. The copyright holder for this preprint (which was not certified by peer review) is the author/funder, who has granted bioRxiv a license to display the preprint in perpetuity. It is made available under aCC-BY 4.0 International license.

Supplement

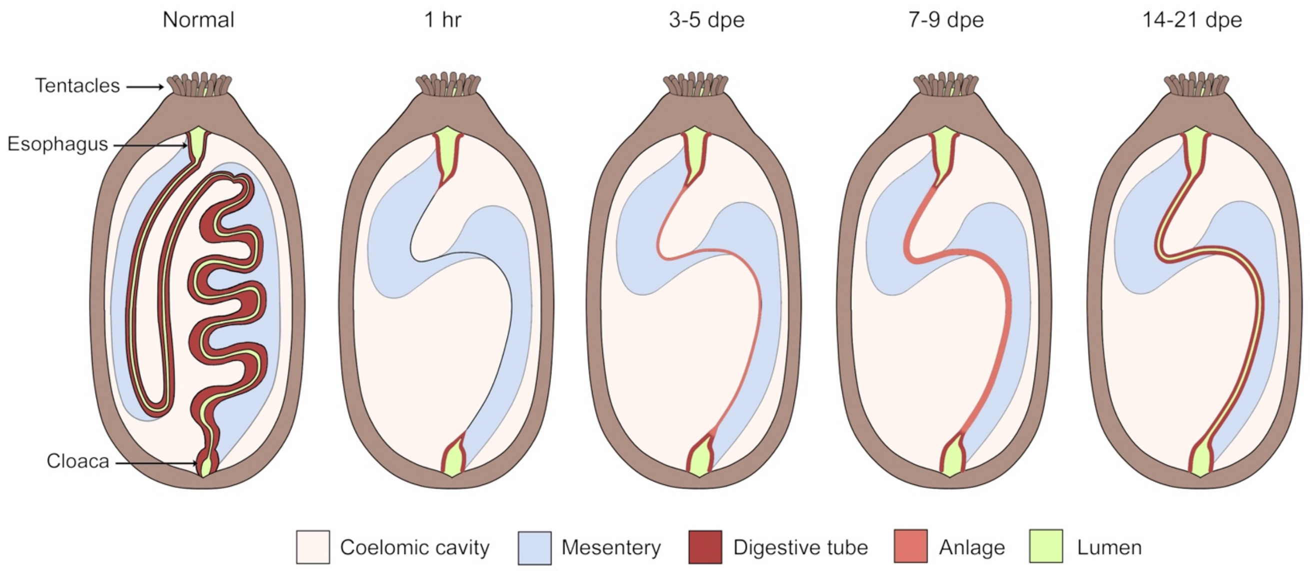

S1 Fig. Overview of the intestinal regeneration process in the sea cucumber *H. glaberrima*. The non-eviscerated (normal) digestive tract is shown as a continuous tube beginning at the mouth, followed by an esophagus which is continuous with the intestine ending in the cloaca. This digestive tract is attached to a mesentery (light blue) which attaches the digestive tract to the body wall. Following evisceration, the tip of the mesentery begins to heal by 24 hrs. After 3 days post evisceration (dpe) the anlage (salmon) begins to form at the free tip of the mesentery and continues growing in the following days. Once the anlage is formed, the lumen develops at around 14-dpe. By 21-dpe the full formed gut lumen (green) can be traced from the esophagus to the cloaca.

bioRxiv preprint doi: https://doi.org/10.1101/2024.07.01.601561; this version posted December 31, 2024. The copyright holder for this preprint (which was not certified by peer review) is the author/funder, who has granted bioRxiv a license to display the preprint in perpetuity. It is made available under aCC-BY 4.0 International license.

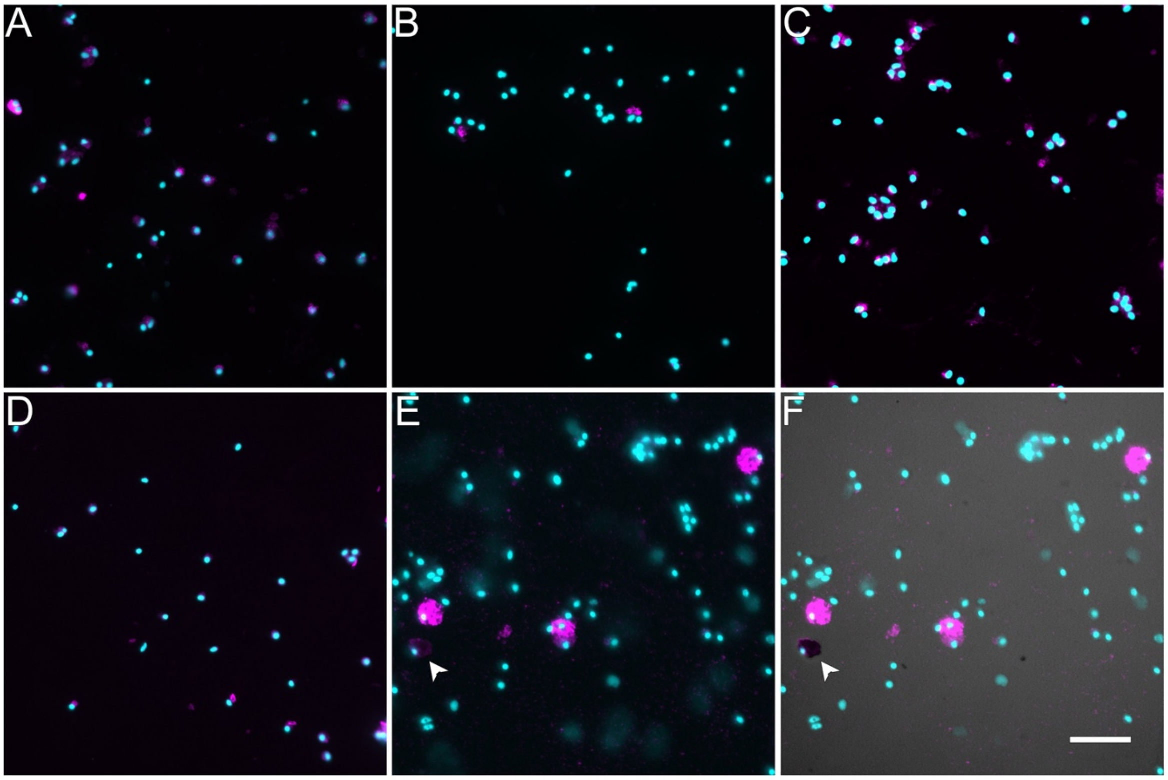

S2 Fig. Labeling of dissociated cell phenotypes with cell markers. Immunocytochemistry, fluorescently labeled phalloidin and Toluidene blue were used to identify various cell populations among the cells dissociated from the mesentery an anlage sample. These include A. a mesenchymal marker (KL4), B. a neuronal marker (RN1), C. a mesothelial marker (Meso1), D. a muscle marker (Phalloidin) and E & F two coelomocyte markers (The antibody SphAA12 and toluidine blue). E. Immunocytochemistry using the SphAA12 antibody F. Overlay of E (UV light) with classical histochemical stain toluidene blue (visible light) identifies a different coelomocyte population (see dark cell on lower left, labeled with arrowhead). Cyan- DAPI, Magenta- antibody or phalloidin marker. Bar= 20um.

bioRxiv preprint doi: https://doi.org/10.1101/2024.07.01.601561; this version posted December 31, 2024. The copyright holder for this preprint (which was not certified by peer review) is the author/funder, who has granted bioRxiv a license to display the preprint in perpetuity. It is made available under aCC-BY 4.0 International license.

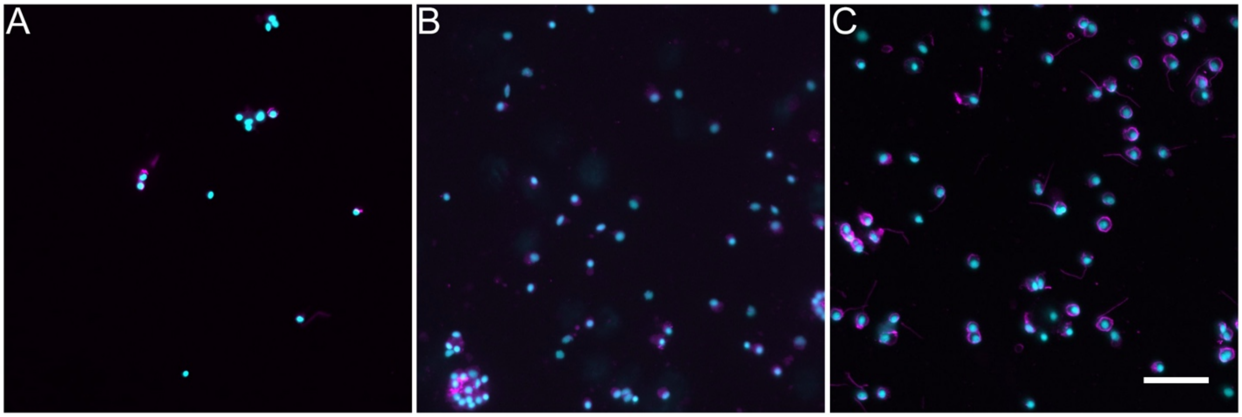

S3 Fig. Immunocytochemical labeling of dissociated cell phenotypes using three different antibodies against tubulin. A. anti-acetylated tubulin labels around 7% of the cells. B. Anti-beta-tubulin labels around 70% of the cells. C. Anti-alpha tubulin labels around 80% of the cells. Cyan- DAPI, Magenta- antibody or phalloidin marker. Bar= 20um.

bioRxiv preprint doi: https://doi.org/10.1101/2024.07.01.601561; this version posted December 31, 2024. The copyright holder for this preprint (which was not certified by peer review) is the author/funder, who has granted bioRxiv a license to display the preprint in perpetuity. It is made available under aCC-BY 4.0 International license.

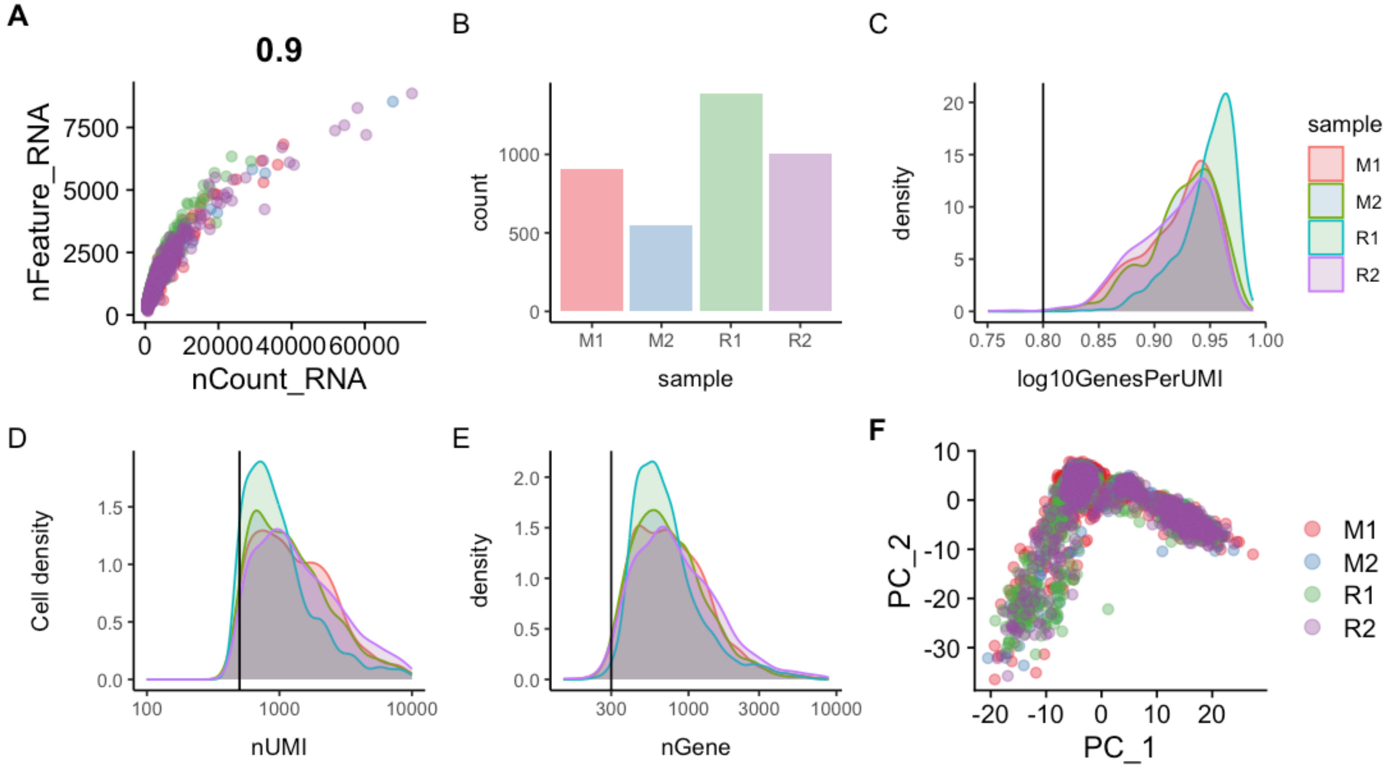

S4 Fig. Quality control assessment of *H. glaberrima* scRNA-seq data. (A) Scatter plot of the correlation of unique genes detected per cell (nFeature_RNA) versus the total number of transcripts (nCount_RNA). Each point in the plot represents a single cell. (B) Bar plot displaying the total number of cells per sample. (C) Density plot of log-transformed genes per UMI (log10GenesPerUMI) showing the sequencing complexity across sample. (D) Density plot of the total number of UMIs (nUMI) per cell showing the distribution of UMI across the four samples. (E) Density plot of the total number of detected genes (nGenes) per cell. Vertical lines in plot C-E represents the minimum threshold cut-off of expected values for each component. (F) Principal component analysis (PCA) plot of the scRNA-seq data after data filtering and integration. M1 and M2 represent mesentery replicates in red and blue, respectively. R1 and R2 represent rudiment/anlage replicates in green and purple, respectively.

bioRxiv preprint doi: https://doi.org/10.1101/2024.07.01.601561; this version posted December 31, 2024. The copyright holder for this preprint (which was not certified by peer review) is the author/funder, who has granted bioRxiv a license to display the preprint in perpetuity. It is made available under aCC-BY 4.0 International license.

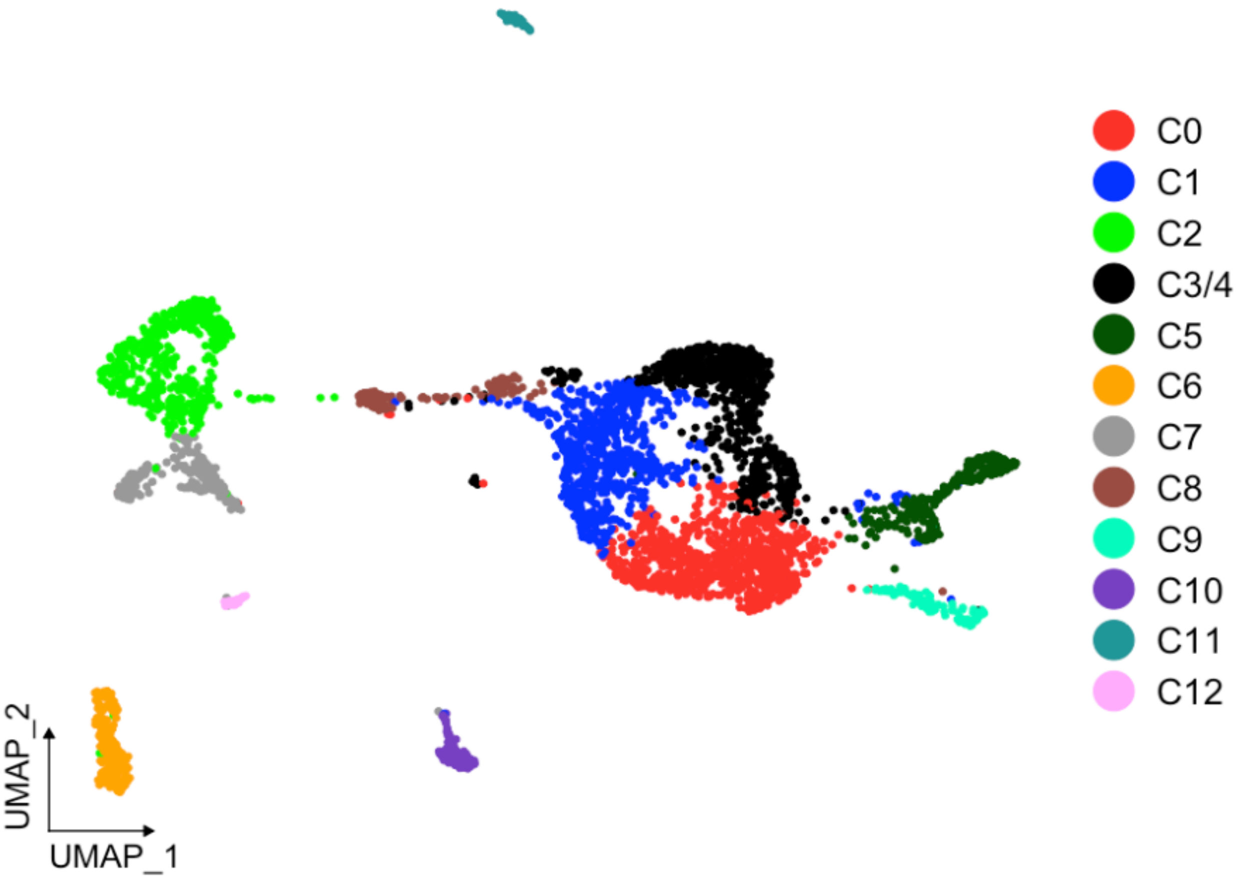

S5 Fig. UMAP of clusters after statistical assessment with scSHC. Results reflect that each of the identified clusters are unique, except for C3 and C4, which it suggests they correspond to a single cluster. All clusters, except for C3/4 (black), are colored as in Fig 1B.

bioRxiv preprint doi: https://doi.org/10.1101/2024.07.01.601561; this version posted December 31, 2024. The copyright holder for this preprint (which was not certified by peer review) is the author/funder, who has granted bioRxiv a license to display the preprint in perpetuity. It is made available under aCC-BY 4.0 International license.

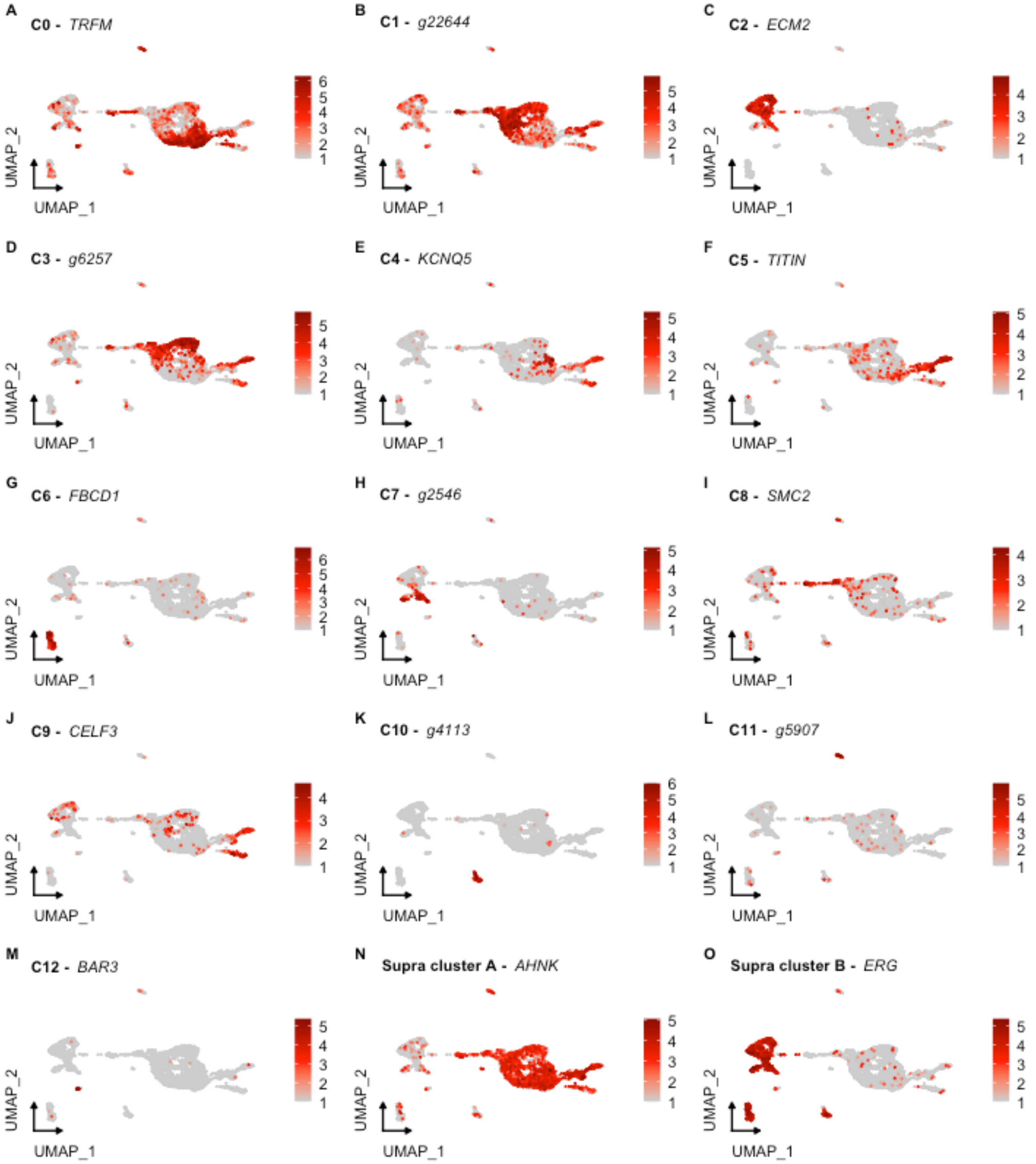

S6 Fig. UMAP visualization of clusters highlighting the expression of their top genes. Each gene corresponds to the top gene of each independent cluster based on the percentage of representation of other clusters. Gene identifiers starting with “g” correspond to uncharacterized gene models of H. glaberrima

bioRxiv preprint doi: https://doi.org/10.1101/2024.07.01.601561; this version posted December 31, 2024. The copyright holder for this preprint (which was not certified by peer review) is the author/funder, who has granted bioRxiv a license to display the preprint in perpetuity. It is made available under aCC-BY 4.0 International license.

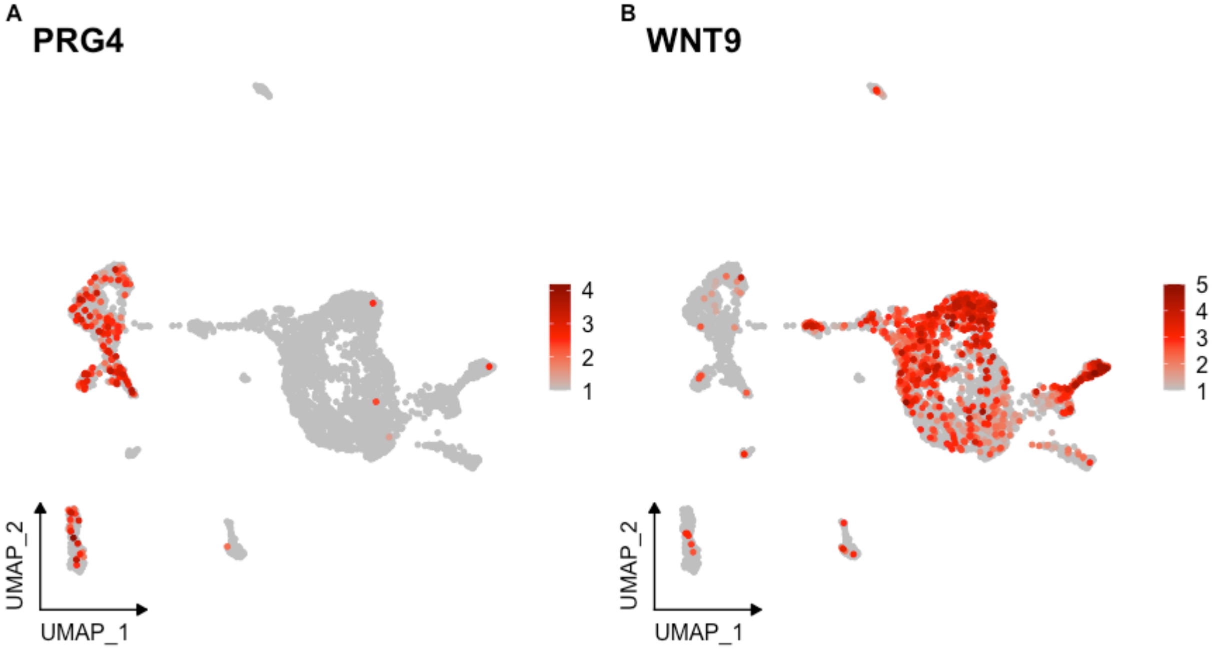

S7 Fig. Expression of marker genes previously documented in the sea cucumber. (A-C) UMAP highlighting the cells expressing (A) Wnt9, (B) SAA, and (C) Proteoglycan-4.

bioRxiv preprint doi: https://doi.org/10.1101/2024.07.01.601561; this version posted December 31, 2024. The copyright holder for this preprint (which was not certified by peer review) is the author/funder, who has granted bioRxiv a license to display the preprint in perpetuity. It is made available under aCC-BY 4.0 International license.

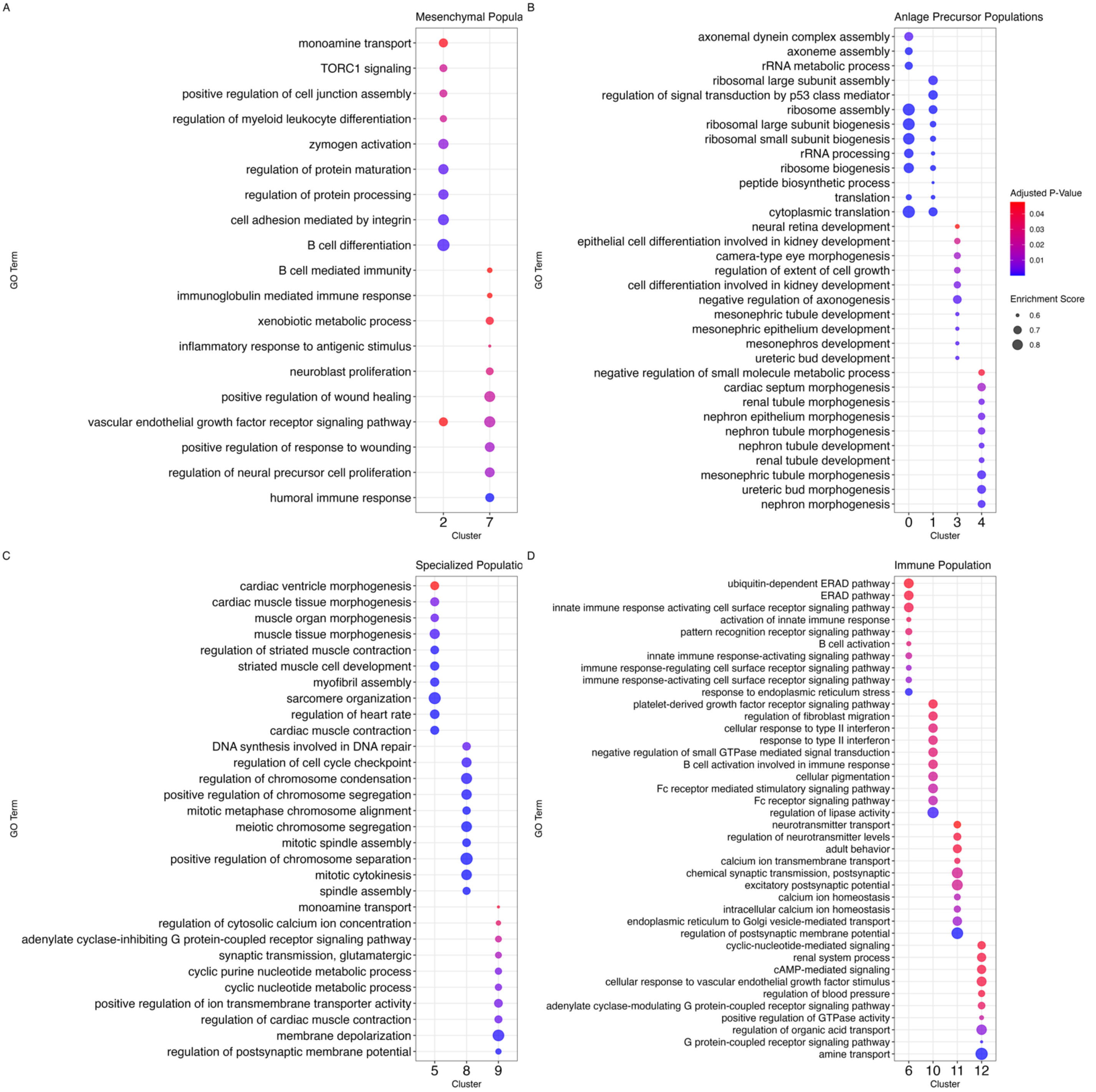

S8 Fig. Enrichment of GO terms across cellular populations of the regenerative intestine of *H. glaberrima*. Top Go enriched terms of biological processes for (A) mesenchymal populations C2 and C7, (B) anlage precursor populations C0, C1, C3, and C4, (C) specialized cell populations C5, C8, C9, and (D) immune populations C6, C10, C11, C12. Dot size corresponds to the enrichment score while the color reflects the adjusted p-value.

bioRxiv preprint doi: https://doi.org/10.1101/2024.07.01.601561; this version posted December 31, 2024. The copyright holder for this preprint (which was not certified by peer review) is the author/funder, who has granted bioRxiv a license to display the preprint in perpetuity. It is made available under aCC-BY 4.0 International license.

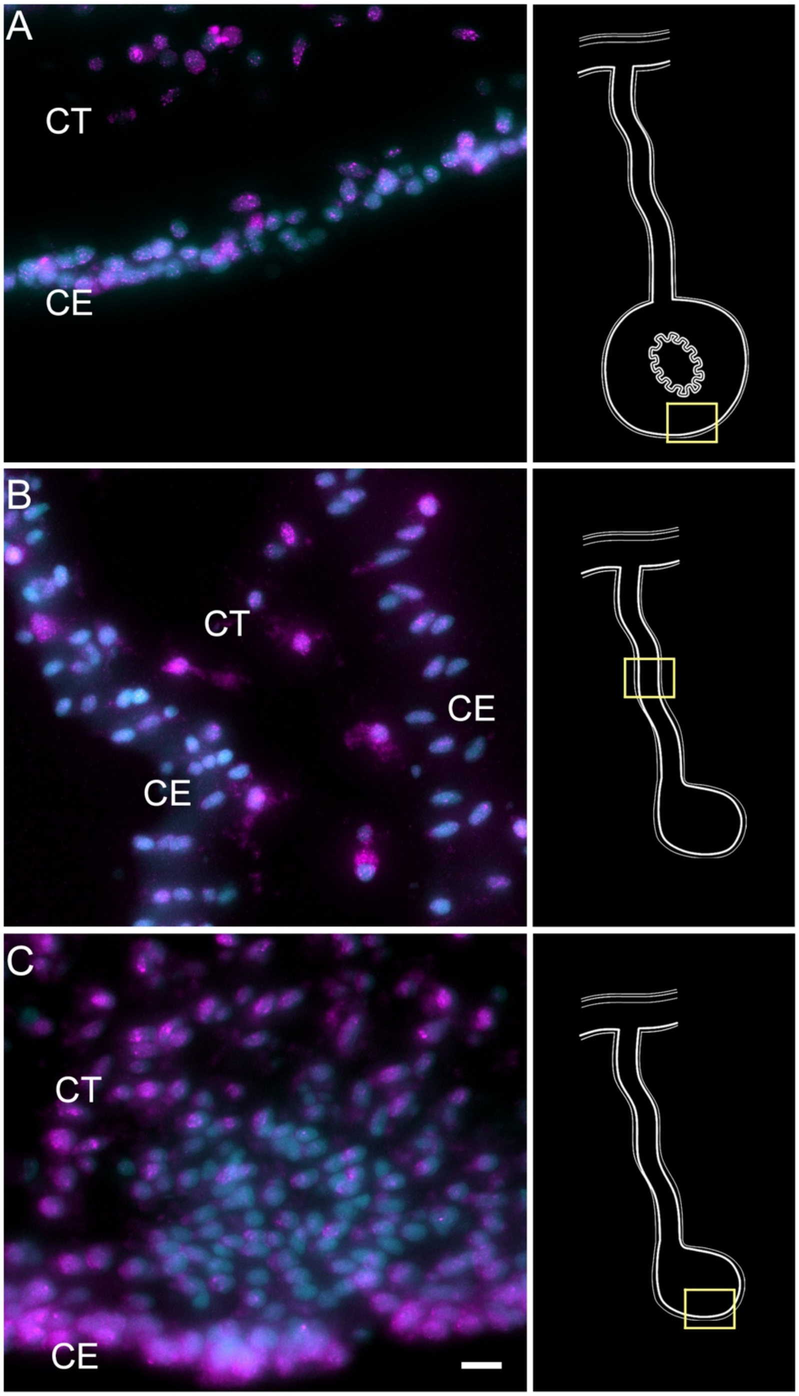

S9 Fig. HCR-FISH for Poly-A mRNA as a positive control. Labeling of *Poly A*-mRNA in the (A) normal intestine, (B) regenerating mesentery, and (C) anlage, provides evidence for distinct labeling of cells in the coelomic epithelia (CE) and connective tissue (CT). Note that some cells express higher intensities of the marker, while others express scarce or no labeling. A particular example is observed in (C) the cells undergoing epithelial-mesenchymal transition in the anlage where little mRNA expression is observed. Insets provide the approximate localization of the cells in the adjacent photos. Cyan- DAPI, Magenta- HCR-FISH. CE- Coelomic epithelium, CT-Connective tissue. Bar= 10µm.

bioRxiv preprint doi: https://doi.org/10.1101/2024.07.01.601561; this version posted December 31, 2024. The copyright holder for this preprint (which was not certified by peer review) is the author/funder, who has granted bioRxiv a license to display the preprint in perpetuity. It is made available under aCC-BY 4.0 International license.

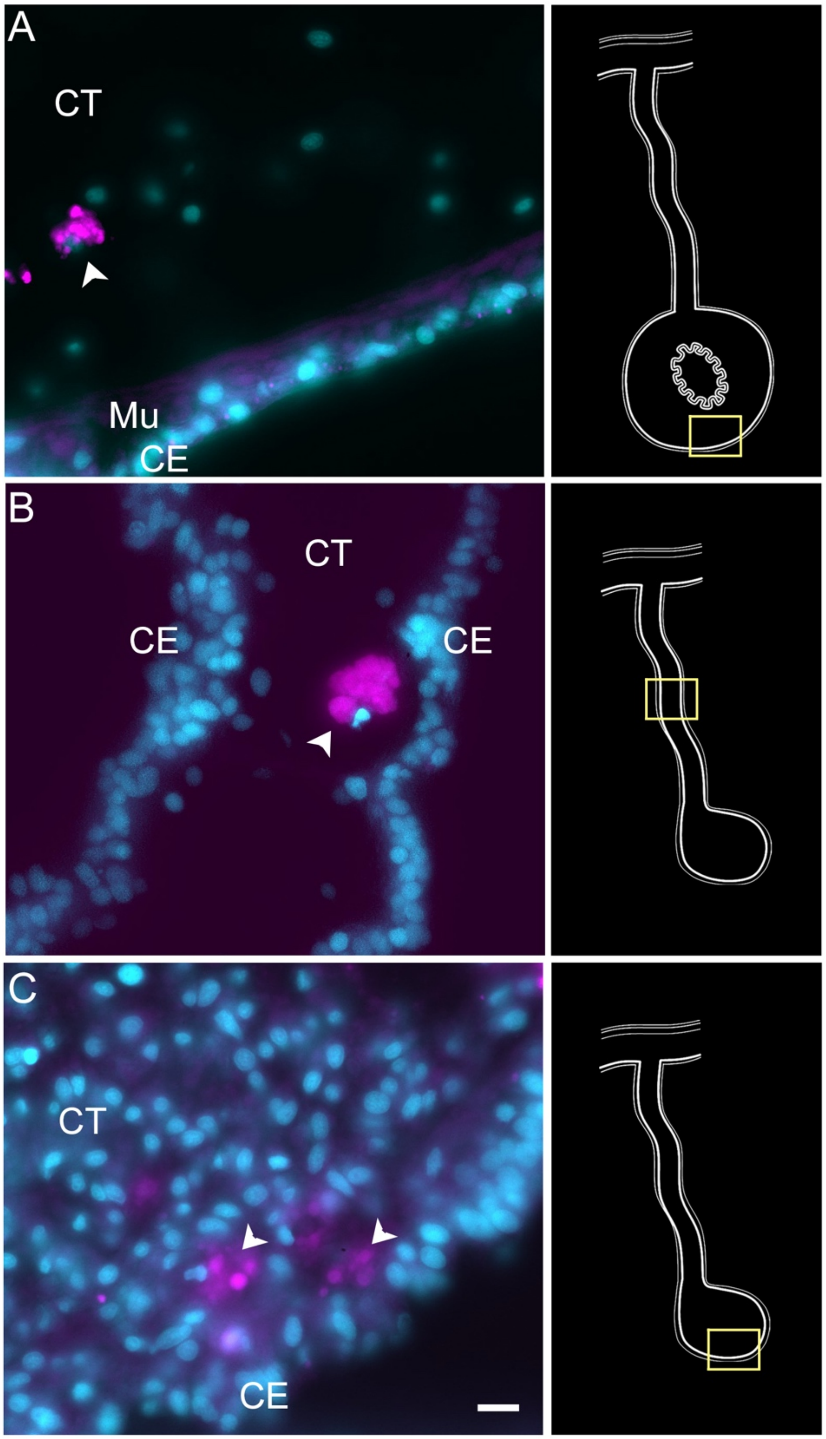

S10 Fig. HCR-FISH negative control. Treatment of tissue sections with only the fluorescent hairpins used for HCR-FISH shows little or no labeling in (A) normal intestine, (B) regenerating mesentery, and (C) anlage. Non-specific labeling is observed in some coelomocytes (arrowheads) found mainly in the connective tissue (CT) and some associated with the coelomic epithelium (CE). Insets provide the approximate localization of the cells in the adjacent photos. Cyan- DAPI, Magenta- HCR-FISH. CE- Coelomic epithelium, CT- Connective tissue. Bar= 10μm.

bioRxiv preprint doi: https://doi.org/10.1101/2024.07.01.601561; this version posted December 31, 2024. The copyright holder for this preprint (which was not certified by peer review) is the author/funder, who has granted bioRxiv a license to display the preprint in perpetuity. It is made available under aCC-BY 4.0 International license.

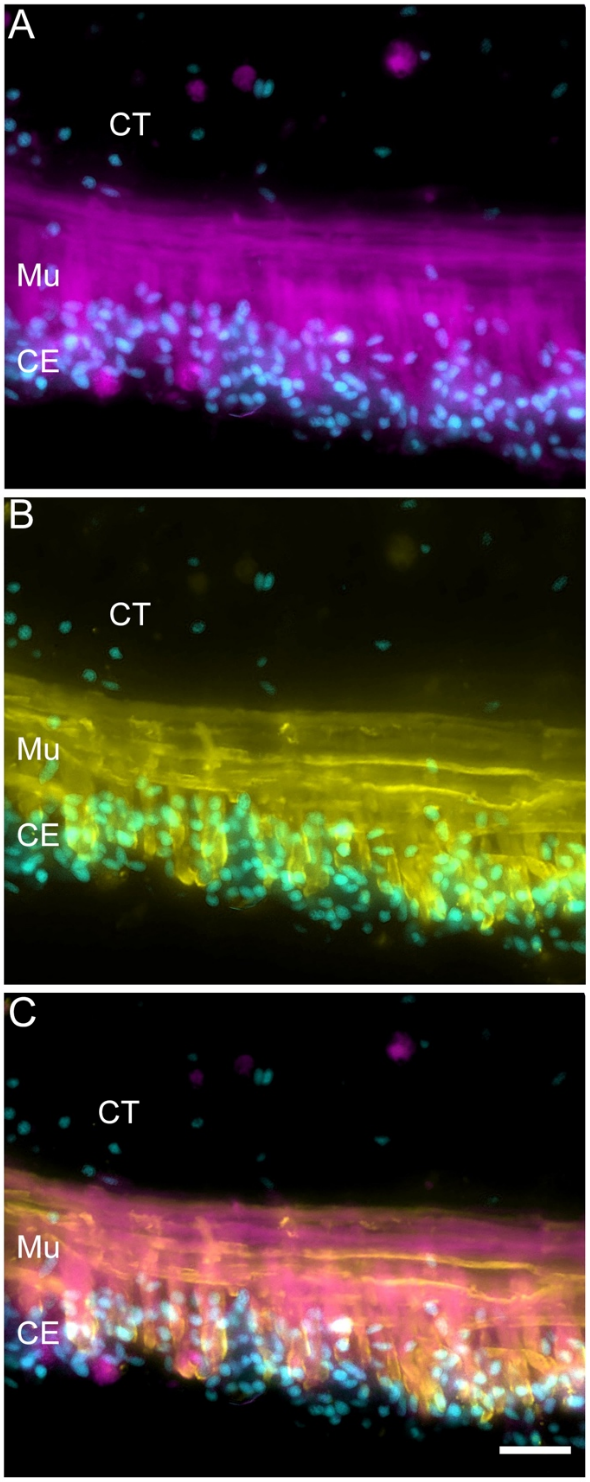

S11 Fig. Double labeling for muscle markers in intestinal muscle cells. Muscle cells in the normal intestine are labeled using (A) fluorescent phalloidin, and (B) muscle-specific antibody (HgM2). (C) The overlay shows that while there is co-expression of both markers in the muscle cells, the markers recognize different structures; phalloidin is known to bind to polymerized actin, while the epitope recognized by the muscle antibody (HgM2), which remains unknown, appears to be associated with the membrane component. Cyan- DAPI, Magenta- fluorescent phalloidin, Yellow- muscle-specific antibody (HgM2). CE- Coelomic epithelium, CT- Connective tissue. Mu- Muscle. Bar= 20µm.

bioRxiv preprint doi: https://doi.org/10.1101/2024.07.01.601561; this version posted December 31, 2024. The copyright holder for this preprint (which was not certified by peer review) is the author/funder, who has granted bioRxiv a license to display the preprint in perpetuity. It is made available under aCC-BY 4.0 International license.

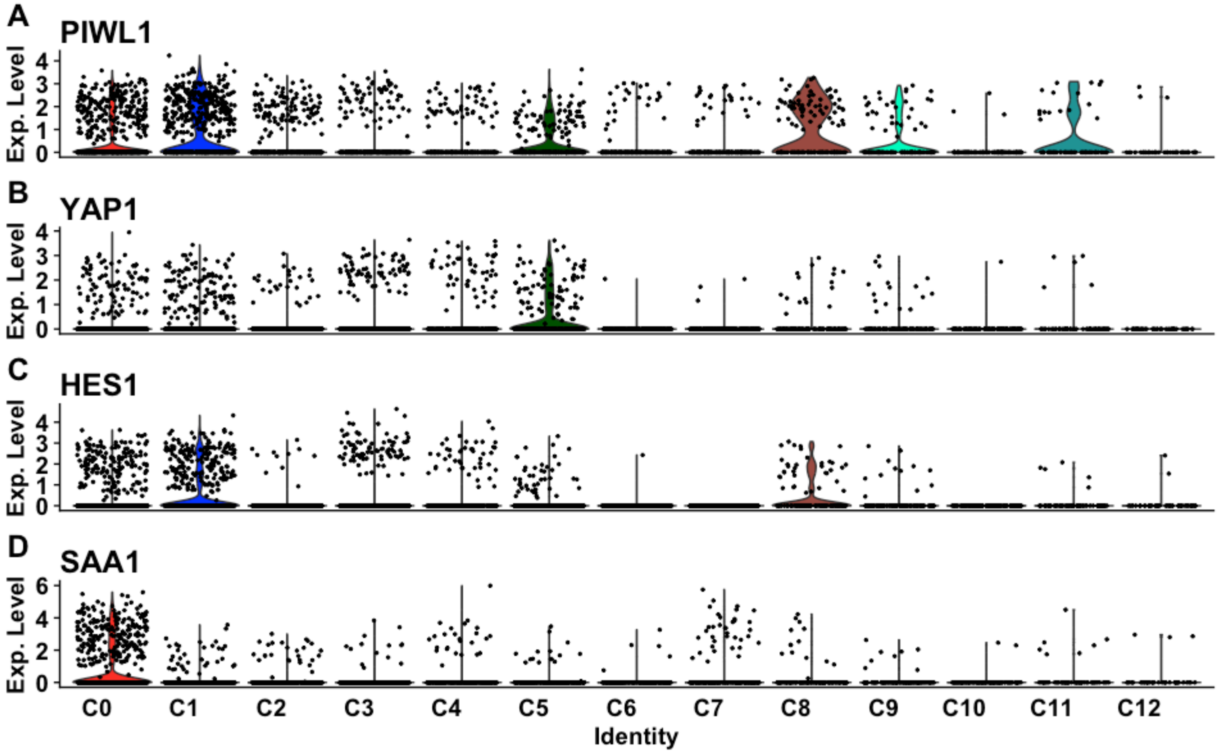

S12 Fig. Expression of marker genes associated with cell types or state. (A-D) Violin plot highlighting the level of expression of (A) PIWL1, (B) YAP1, (C) HES1, and (D) SAA1 across clusters.

bioRxiv preprint doi: https://doi.org/10.1101/2024.07.01.601561; this version posted December 31, 2024. The copyright holder for this preprint (which was not certified by peer review) is the author/funder, who has granted bioRxiv a license to display the preprint in perpetuity. It is made available under aCC-BY 4.0 International license.

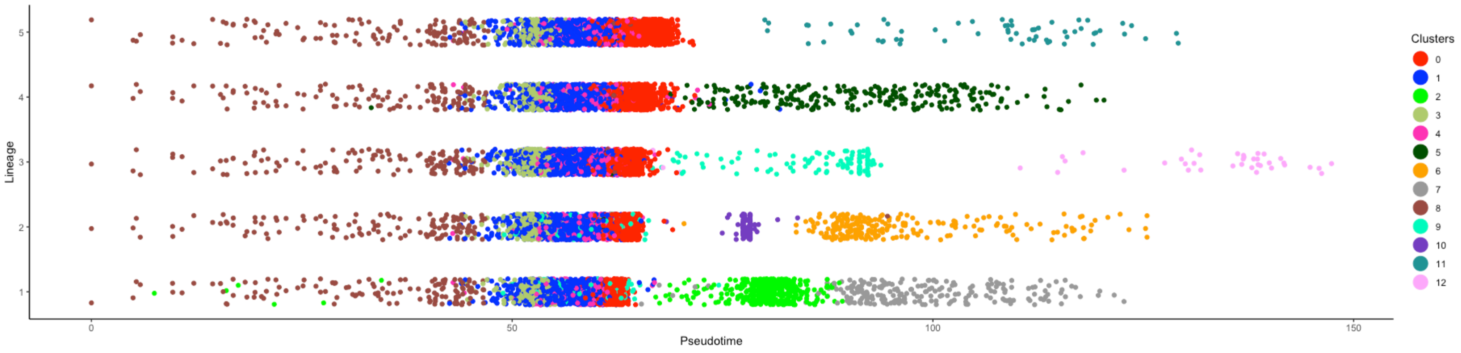

S13 Fig. Pseudotime analysis of all clusters within the 9dpe intestinal regeneration dataset. Each color reflects the corresponding cluster form C0-C12. The x-axis represents slingshot pseudotime and y-axis the corresponding lineage.

S14 Table. Antibody/Markers used for immune- and cytochemical labeling of dissociated cell suspension.

**Antibody/Marker**	**Labeling**	**Product No.**	**Reference**
Ab Sph AA12	Coelomocyte	-	Ramírez-Gómez et al., 2010
Ab RN1	Neuronal	-	Nieves-Ríos et al., 2020
Ab GFS	Neuronal	-	Diaz-Balzac et al., 2007
Ab KL14	Mesenchyme	-	In preparation
Ab MESO	Mesothelial cells	-	García-Arrarás et al., 2011
Ab HgM2	Muscle	-	In preparation
Ab beta tubulin	Neuronal/Cilia	Sigma T5293	Tossas et al., 2014
Ab Acetylated tubulin	Cellular	Sigma T6793	Tossas et al., 2014
Ab alpha tubulin	Cellular	Sigma T5168	Tossas et al., 2014
Fluorescent Phalloidin	Muscle fibers	Sigma P1951	García-Arrarás et al., 2011; Murray and García-Arrarás et al., 2004
Toluidine Blue	Cell granular content	Sigma T3260	San Miguel-Ruiz & Garcia-Arraras, 2007

S15 Table. General statistics of scRNA-seq data after mapping with Cell Ranger.

**Description**	**Mesentery 1**	**Mesentery 2**	**Anlage 1**	**Anlage 2**
Estimated Number of Cells	903	549	1,388	1,004
Number of Reads	86,939,451	120,621,009	52,320,210	151,638,195
Mean Reads per Cell	96,278	237,925	37,695	151,034
Median Genes per Cell	725	687	643	752
Total Genes Detected	20,193	18,707	19,612	20,436
