# Supplementary material for: Single-cell RNA sequencing of the holothurian regenerating intestine reveals the pluripotency of the coelomic epithelium
Source: bioRxiv. 2024 Jul 4:2024.07.01.601561. Preprint. [Version 1] doi: 10.1101/2024.07.01.601561 (PMC11244903; doi:10.1101/2024.07.01.601561)
Supplement: Supplementary file 1 [file NIHPP2024.07.01.601561v1-supplement-1.pdf]

bioRxiv preprint doi: https://doi.org/10.1101/2024.07.01.601561; this version posted July 5, 2024. The copyright holder for this preprint (which was not certified by peer review) is the author/funder, who has granted bioRxiv a license to display the preprint in perpetuity. It is made available under aCC-BY 4.0 International license.

Supplement

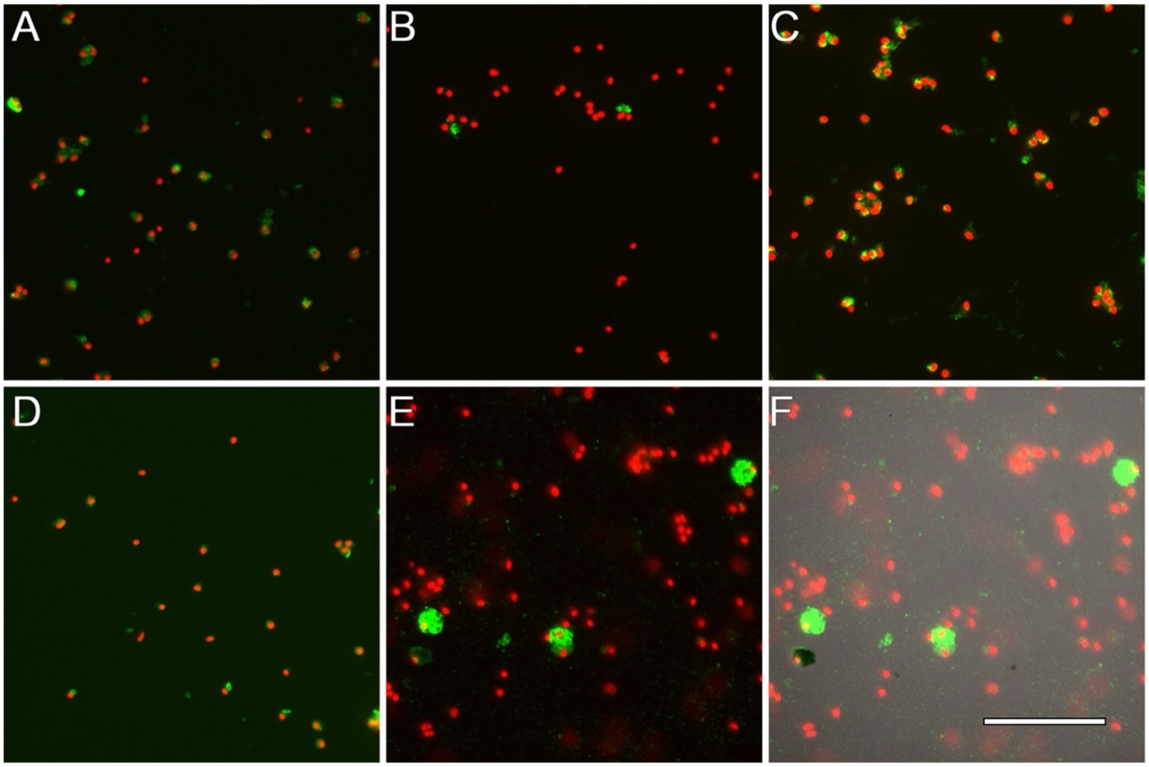

S1 Fig. Labeling of dissociated cell phenotypes with cell markers. Immunocytochemistry, fluorescently labeled phalloidin and Toluidene blue were used to identify various cell populations among the cells dissociated from the mesentery an anlage sample. These include A. a mesenchymal marker (KL4), B. a neuronal marker (RN1), C. a mesothelial marker (Meso1), D. a muscle marker (Phalloidin) and E & F two coelomocyte markers (The antibody SphAA12and toluidine blue). E. Immunocytochemistry using the SphAA12 antibody F. Overlay of toluidene blue labeling (see dark cell on lower left) identifies a different coelomocyte population.

bioRxiv preprint doi: https://doi.org/10.1101/2024.07.01.601561; this version posted July 5, 2024. The copyright holder for this preprint (which was not certified by peer review) is the author/funder, who has granted bioRxiv a license to display the preprint in perpetuity. It is made available under aCC-BY 4.0 International license.

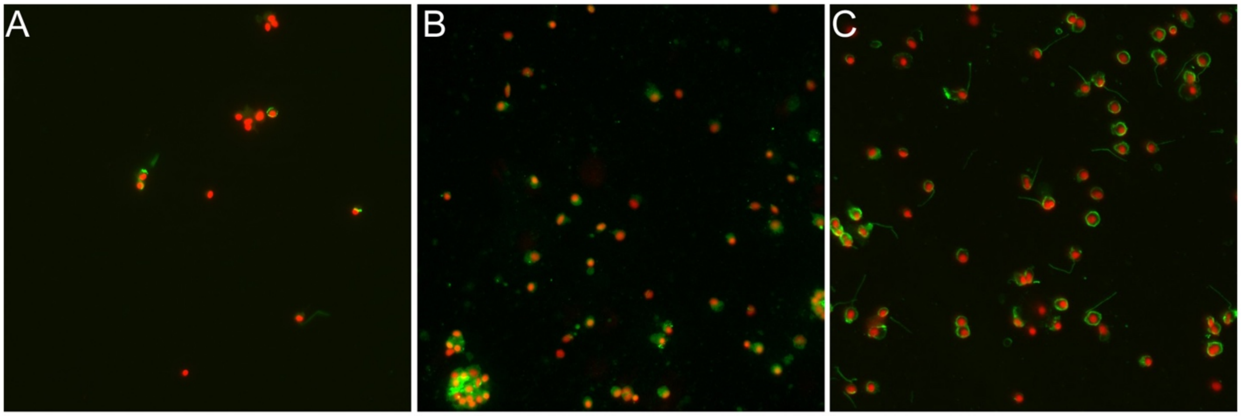

S2 Fig. Immunocytochemical labeling of dissociated cell phenotypes using three different antibodies against tubulin. A. anti-acetylated tubulin labels around 7% of the cells. B. Anti-beta-tubulin labels around 70% of the cells. C. Anti-alpha tubulin labels around 80% of the cells.

bioRxiv preprint doi: https://doi.org/10.1101/2024.07.01.601561; this version posted July 5, 2024. The copyright holder for this preprint (which was not certified by peer review) is the author/funder, who has granted bioRxiv a license to display the preprint in perpetuity. It is made available under aCC-BY 4.0 International license.

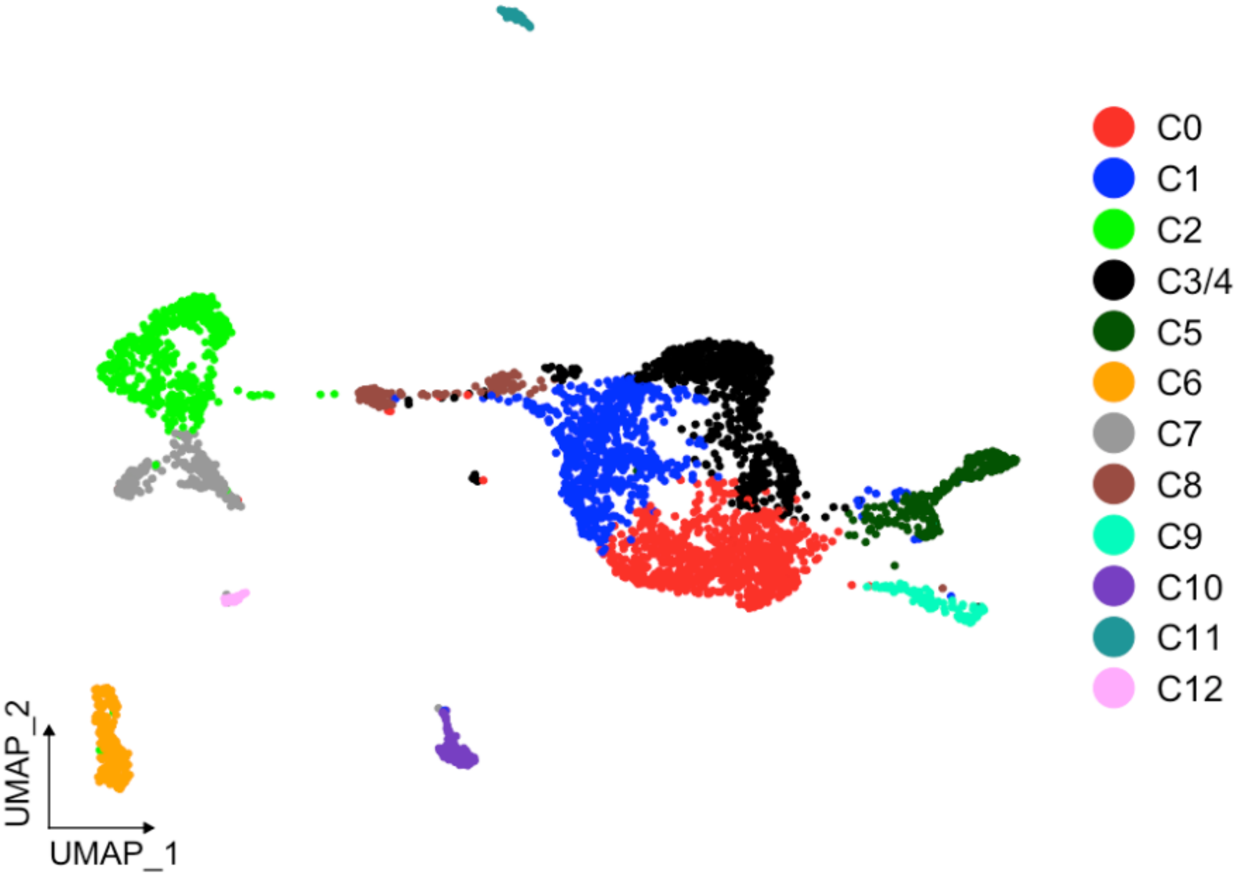

S3 Fig. UMAP of clusters after statistical assessment with scSHC. Results reflect that each of the identified clusters are unique, except for C3 and C4, which it suggests they correspond to a single cluster. All clusters, except for C3/4 (black), are colored as in Fig 1B.

bioRxiv preprint doi: https://doi.org/10.1101/2024.07.01.601561; this version posted July 5, 2024. The copyright holder for this preprint (which was not certified by peer review) is the author/funder, who has granted bioRxiv a license to display the preprint in perpetuity. It is made available under aCC-BY 4.0 International license.

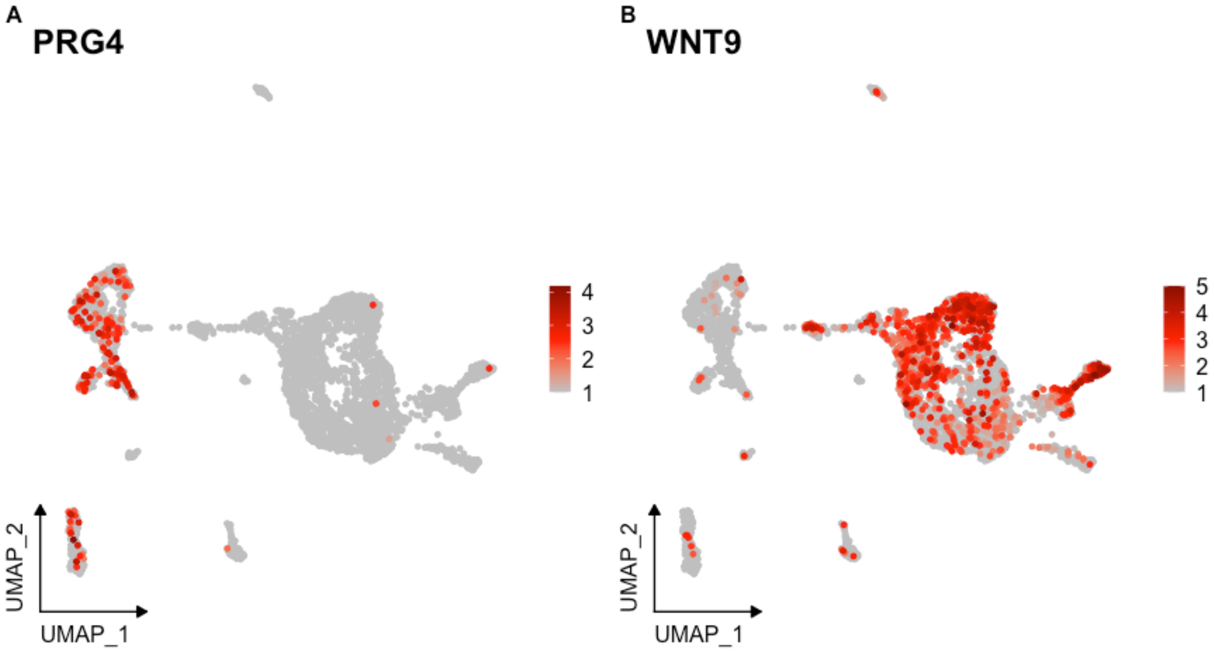

S4 Fig. Expression of marker genes previously documented in the sea cucumber. (A-C) UMAP highlighting the cells expressing (A) Wnt9, (B) SAA, and (C) Proteoglycan-4.

bioRxiv preprint doi: https://doi.org/10.1101/2024.07.01.601561; this version posted July 5, 2024. The copyright holder for this preprint (which was not certified by peer review) is the author/funder, who has granted bioRxiv a license to display the preprint in perpetuity. It is made available under aCC-BY 4.0 International license.

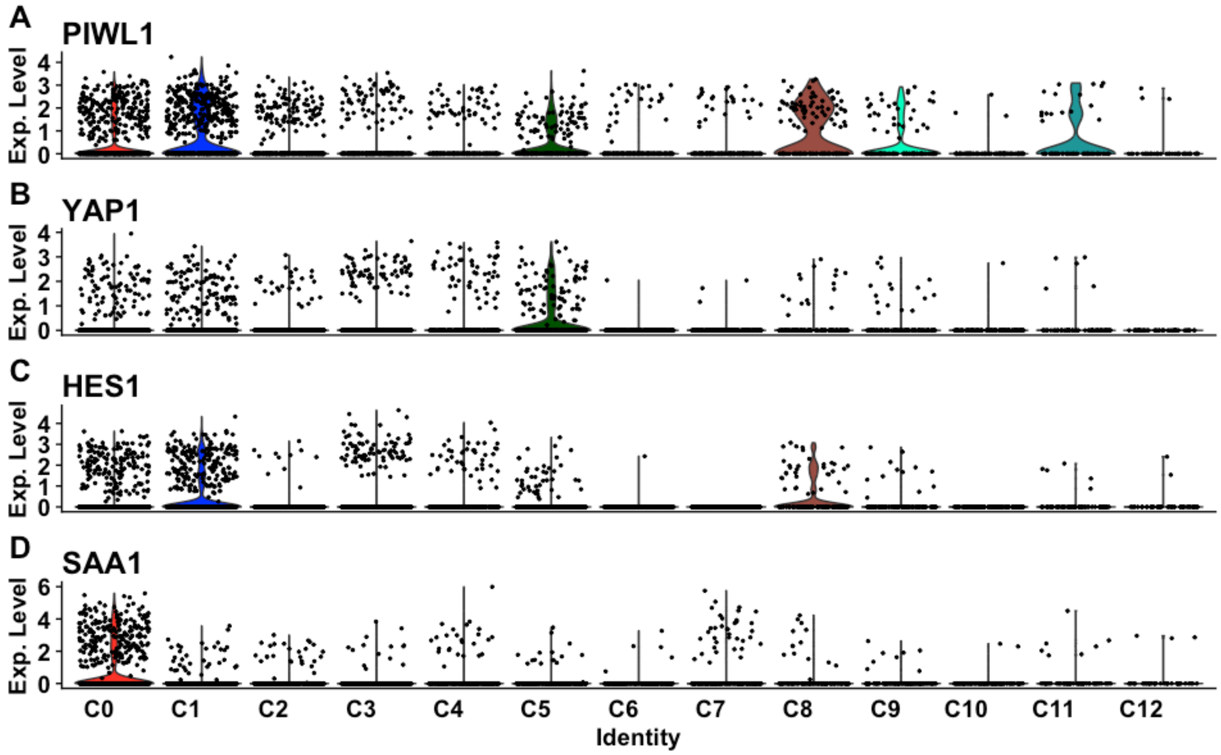

S5 Fig. Expression of marker genes associated with cell types or state. (A-D) Violin plot highlighting the level of expression of (A) PIWL1, (B) YAP1, (C) HES1, and (D) SAA1 across clusters.

bioRxiv preprint doi: https://doi.org/10.1101/2024.07.01.601561; this version posted July 5, 2024. The copyright holder for this preprint (which was not certified by peer review) is the author/funder, who has granted bioRxiv a license to display the preprint in perpetuity. It is made available under aCC-BY 4.0 International license.

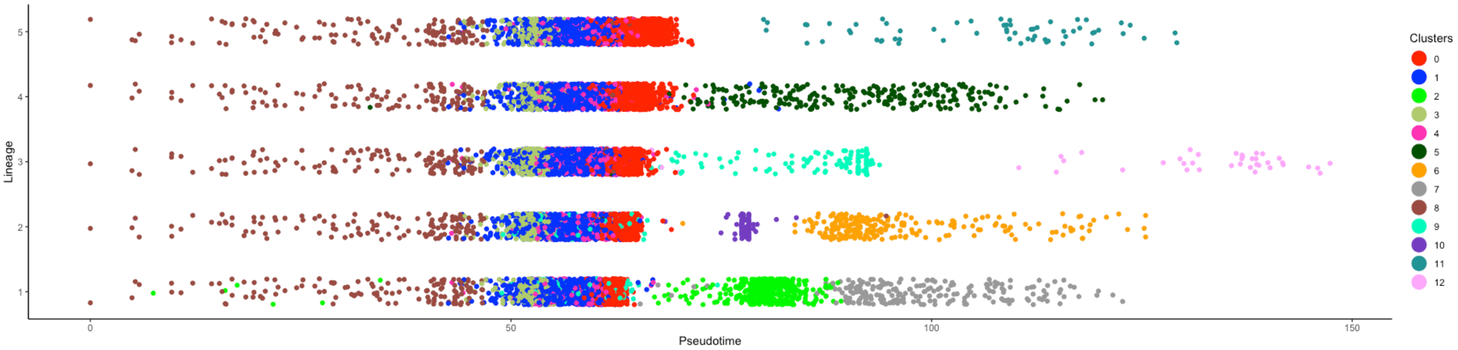

S6 Fig. Pseudotime analysis of all clusters within the 9dpe intestinal regeneration dataset. Each color reflects the corresponding cluster form C0-C12. The x-axis represents slingshot pseudotime and y-axis the corresponding lineage.

bioRxiv preprint doi: https://doi.org/10.1101/2024.07.01.601561; this version posted July 5, 2024. The copyright holder for this preprint (which was not certified by peer review) is the author/funder, who has granted bioRxiv a license to display the preprint in perpetuity. It is made available under aCC-BY 4.0 International license.

S7 Table. Antibody/Markers used for immune- and cytochemical labeling of dissociated cell suspension.

Antibody/Marker	Labeling	Product No.	Reference
Ab Sph AA12	Coelomocyte	-	Ramírez-Gómez et al., 2010
Ab RN1	Neuronal	-	Nieves-Ríos et al., 2020
Ab GFS	Neuronal	-	Diaz-Balzac et al., 2007
Ab KL14	Mesenchyme	-	In preparation
Ab MESO	Mesothelial cells	-	García-Arrarás et al., 2011
Ab beta tubulin	Neuronal/Cilia	Sigma T5293	Tossas et al., 2014
Ab Acetylated tubulin	Cellular	Sigma T6793	Tossas et al., 2014
Ab alpha tubulin	Cellular	Sigma T5168	Tossas et al., 2014
Fluorescent Phalloidin	Muscle fibers	Sigma P1951	García-Arrarás et al., 2011; Murray and García-Arrarás et al., 2004
Toluidine Blue	Nuclei		
